# Visual attention and inhibitory control in children, teenagers and adults with autism without intellectual disability: results of oculomotor tasks from a 2-year longitudinal follow-up study (InFoR)

**DOI:** 10.1186/s13229-021-00474-2

**Published:** 2021-11-13

**Authors:** Anouck Amestoy, Etienne Guillaud, Giulia Bucchioni, Tiziana Zalla, Daniel Umbricht, Christopher Chatham, Lorraine Murtagh, Josselin Houenou, Richard Delorme, Myriam Ly-Le Moal, Marion Leboyer, Manuel Bouvard, Jean-René Cazalets

**Affiliations:** 1 CNRS, Aquitaine Institute for Cognitive and Integrative Neuroscience, INCIA, UMR 5287, Université de Bordeaux, 33000 Bordeaux, France; 2 iBrain, UMR 1253 Inserm, Université de Tours, 2 Boulevard Tonnellé, 37044 Tours Cedex, France; 3 Roche Pharma Research and Early Development, Roche Innovation Center Basel, F. Hoffmann-La Roche Ltd., Grenzacherstrasse 124, 4070 Basel, Switzerland; 4 Laboratoire de NeuroPsychiatrie translationnelle, INSERM, U955, IMRB, Créteil, France; 5 Fondation FondaMental, Créteil, France; 6 NeuroSpin, UNIACT Lab, Equipe de psychiatrie, Commissariat à l’énergie atomique, Saclay, Gif-sur-Yvette, France; 7 Institut Pasteur, Paris, France; 8 AP-HP, DMU IMPACT, Psychiatry and Addictology Department, Mondor University Hospital, Université Paris Est Créteil, Créteil, France; 9 Institut Roche, Tour horizons- Bureau 18M3, Roche S.A.S., 30, cours de l’île Seguin, 92650 Boulogne-Billancourt, France; 10 centre hospitalier Charles-Perrens, Pôle universitaire de psychiatrie de l’enfant et de l’adolescent, 121, rue de la Béchade, CS 81285, 33076 Bordeaux Cedex, France; 11 Institut Jean Nicod, ENS, Paris, France

**Keywords:** Autism spectrum disorders (ASD), Oculomotor behavior, Eye-tracking, Gap–Overlap–Step tasks, Antisaccade task, Inhibitory control, Attention shifting

## Abstract

**Background:**

Inhibitory control and attention processing atypicalities are implicated in various diseases, including autism spectrum disorders (ASD). These cognitive functions can be tested by using visually guided saccade-based paradigms in children, adolescents and adults to determine the time course of such disorders.

**Methods:**

In this study, using Gap, Step, Overlap and Antisaccade tasks, we analyzed the oculomotor behavior of 82 children, teenagers and adults with high functioning ASD and their peer typically developing (TD) controls in a two-year follow-up study under the auspices of the **InFoR**-**Autism** project. Analysis of correlations between oculomotors task measurements and diagnostic assessment of attentional (ADHD-RS and ADHD comorbidity indices) and executive functioning (BRIEF scales) were conducted in order to evaluate their relationship with the oculomotor performance of participants with ASD.

**Results:**

As indicated by the presence of a Gap and Overlap effects in all age groups, the oculomotor performances of ASD participants showed a preserved capability in overt attention switching. In contrast, the difference in performances of ASD participants in the Antisaccade task, compared to their TD peers, indicated an atypical development of inhibition and executive functions. From correlation analysis between our oculomotor data and ADHD comorbidity index, and scores of attention and executive function difficulties, our findings support the hypothesis that a specific dysfunction of inhibition skills occurs in ASD participants that is independent of the presence of ADHD comorbidity.

**Limitations:**

These include the relatively small sample size of the ASD group over the study’s two-year period, the absence of an ADHD-only control group and the evaluation of a TD control group solely at the study’s inception.

**Conclusions:**

Children and teenagers with ASD have greater difficulty in attention switching and inhibiting prepotent stimuli. Adults with ASD can overcome these difficulties, but, similar to teenagers and children with ASD, they make more erroneous and anticipatory saccades and display a greater trial-to-trial variability in all oculomotor tasks compared to their peers. Our results are indicative of a developmental delay in the maturation of executive and attentional functioning in ASD and of a specific impairment in inhibitory control.

**Supplementary Information:**

The online version contains supplementary material available at 10.1186/s13229-021-00474-2.

## Background

Autism spectrum disorders (ASD) are severe and lifelong neurodevelopmental disorders, characterized by difficulties in social interactions, verbal and nonverbal communication, as well as sensory abnormalities, stereotypic repetitive behavioral patterns and limited interests and activities [1]. Perceptual abnormalities are also frequently observed in individuals with ASD, and these sensory modulation atypicalities are correlated with the severity of social difficulties [1, 2]. Since the major and primary atypicalities of individuals with ASD lie in social skills and communication, most studies on eye movements have focused on scan patterns of social scenes and facial expressions. Nevertheless, social processing impairment may not be entirely social in origin, but in part also a visual perceptual impairment, which may be attentional in nature. In addition, oculomotor abnormalities that have been described in patients with ASD ([3, 4, for meta-analysis, see 5–8] suggest general attentional and executive dysfunctions. A growing body of research has therefore focused on visual perceptual skills and oculomotor behaviors involving non-social stimuli in ASD participants, which implicate visual attention processes and lower-level visual motor control, using various visually guided saccade (VGS) paradigms.

Attention networks include those responsible for alerting, orienting and executive control [9]. Executive functions include, among others, working memory, inhibitory control, directed attention, cognitive flexibility and initiation of action [10, 11]. The use of such cognitive processes is highly demanding, and atypicalities in these processes have been identified in various pathologies [12], including autism [13, 14]. In ASD, several studies have investigated the impact of executive and attentional function difficulties by means of assessing oculomotor task performances [for review, see 15, 16]. For example, previous studies on ASD have described early difficulties in the orientation of visual attention toward social and non-social stimuli in infants (< 26 months old) [17–19], children and teenagers [20], as well as oculomotor atypicalities in attentional flexibility and in disengagement of attention in infants [21–23].

**Fig. 1:**
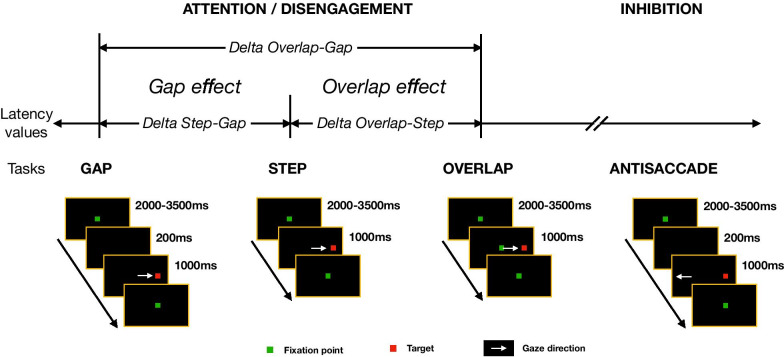
Experimental design. From left to right: Gap, Step, Overlap and Antisaccade tasks. As indicated by the white arrows (in lower panels), in the first three tasks, after an initial center screen fixation (green boxes), participants had to move their gaze toward a peripheral target (red squares) as soon as it appeared on the screen. The central anchoring point (green square) disappeared prior to (Gap task) or coincident with (Step task, i.e., 0-Gap) the peripheral target’s appearance, or it remained on screen along with target in the Overlap task. In the Antisaccade task, participants had to move their eyes in the opposite direction to that of the target. The Gap, Step and Overlap tasks allow the evaluation of attention disengagement by means of the Gap (Gap latency < Step latency) and Overlap effects (Step latency < Overlap latency). The Antisaccade task allows assessment of inhibitory control (Gap latency < Antisaccade latency)

Computer-based VGS paradigms using exogenous cues to measure oculomotor control, attention disengagement and inhibition [16, 24–27]. Various other studies have suggested that under experimental conditions, different specific oculomotor tasks elicit distinct attentional and executive function levels and vary in demands placed on attention and inhibition [16, 26, 28–32]. The various VGS paradigms include the Gap, Step and Overlap tasks in which a subject is required to disengage and shift attention from a central fixation point to a peripheral target that appears in 3 different ways depending on the task (Fig. 1). The differences in saccade latency values between these tasks allow exploration of the so-called Gap and Overlap effects [see Methods for details 22, 24, 33–35]. Available literature on the hypothesis of impaired oculomotor control in ASD has provided several lines of evidence for an intrinsic control of reflexive saccades, albeit with reduced accuracy [36, 37] and a diminished ability of oculomotor behavior to correct for saccadic errors [5, 38, 39], although other authors have suggested the occurrence of specific, more extensive atypicalities [16, 18, 23, 40].

According to Keehn et al. [15], the ASD-associated atypicality could mainly be related to issues in orienting/attentional network functioning (i.e., the selection of information from sensory input) and could also be explained by atypicalities in the executive control network. Inhibitory control of executive functions (i.e., the capacity to suppress inappropriate automatic responses in order to promote an appropriate response to a cognitive goal [for review and theoretical background of inhibition development 41–43] can be studied using the Antisaccade task (gaze fixating on the mirrored position of a target) that requires overriding a prepotent response [for review, see 44]. In the Antisaccade task, an atypical inhibition of reflexive responses will result in a high number of reflexive saccades toward the target (prosaccades), whereas difficulties in the generation of voluntary movements will result in a low number of antisaccades (i.e., toward the opposite side). Such an impairment of inhibitory control of executive functions has also been widely reported in autism, both in adults [45, 46] and children [47–50], and can be diagnosed as early as the second year after birth [51]. Moreover, it appears to be related to repetitive behaviors, which are a core symptom in ASD individuals [24, 46]. Previous studies reported that subjects with ASD perpetuate a greater number of errors compared to TD subjects in Antisaccade tasks [5, 24, 45, 52–54], but there is still disagreement about the maturation of inhibitory control in ASD subjects compared to TD controls. For instance, Luna et al. [55] suggested that an equivalent improvement of inhibitory control occurs during development, whereas Padmanabhan et al. [56] and Schmitt et al. [49] reported an attenuated age-related improvement of inhibitory control in ASD subjects.

Although these studies indicated ASD age-related improvements of attention and inhibition, the results remain inconsistent and the maturation of these functions has not been systematically assessed over time via age group comparisons. Various discordant findings in the literature have been reported for oculomotor behavior in subjects with ASD. The recent meta-analysis of 27 studies has underlined the wide range of different types of basic oculomotor tasks employed to assess subjects with ASD [5]. The results suggested that individuals with ASD show relatively few oculomotor deficiencies and a high degree of heterogeneity and variability, but make significantly more errors than control subjects, especially in the Antisaccade task that focuses on volitional saccades [5]. This in turn pointed to specific difficulties in inhibiting a rapid response in ASD subjects, although the main limitations of all these studies were the large age range of participants, the lack of comparisons between age groups within the same test paradigm, the absence of IQ-matched controls, and the heterogeneity of the paradigms used to induce saccades.

To address these issues, the present study aimed to investigate the maturation and stability of the two cognitive functions underlying oculomotor behaviors: (1) *visual attention* (focusing on the capacity for attentional shifting and disengagement) through the comparison of Gap and Overlap saccadic latencies with Step latencies and (2) *inhibitory control* by comparing response latencies in the Gap and Antisaccade tasks in high-functioning ASD participants at three consecutive time points (T0, then at 1 and 2 years later) and in three different age groups (children, teenagers and adults), compared to their TD peers. The influence of other executive functions was tested by making correlations with clinical data. Moreover, correlations with the assessment of attention deficit/hyperactivity disorder (ADHD) symptoms and ADHD comorbidity were also examined in our study.

## Methods

### Participants

ASD and TD participants were recruited in three French centers (Bordeaux, Paris, Créteil) with recognized expertise in ASD and coordinated by the Fondation FondaMental via the so-called InFoR project, which was a 2-year, multicentered, longitudinal and non-drug involvement program comprising 97 participants with ASD and 49 control participants (see Table 1 for a detailed description of participant profiles). These two main cohorts, termed “All ASD” and “All TD” groups, respectively, were subdivided into 3 different age categories: children (6–11 years), teenagers (12–17 years) and adults (> 18 years). The groups were matched for age and full-scale IQ. Two-way ANOVAs (Clinical group × Gender) indicated that whatever the age group, there was no significant difference between ASD and TD either in terms of age (TD vs ASD, adults: *p* = 0.94; teenagers: *p* = 0.78; children: *p* = 0.93) or IQ (TD vs ASD, adults: *p* = 0.49; teenagers: *p* = 0.27; children: *p* = 0.24). Exclusion criteria for all participants were: (1) age < 6 or > 56 years old; (2) full IQ score < 70. Additional exclusion criteria for the control group were the presence of a previously diagnosed genetic disorder or any neurological, epilepsy or psychiatric history, as verified with DIGS [Diagnostic Interview for Genetic Studies, 57] for adults or Kiddie-SADS [Diagnostic Interview for Genetic Studies, 58] for children. All participants reported normal or corrected-to-normal vision. Our study was approved by local ethics committees (AFSSAPS B80738-70) and registered in the public trial registry (NCT02628808). All adult participants or parents of juvenile participants were required to provide formal written consent before inclusion in this study.

**Table 1:** Participant profiles at study onset (time point T0)

	*N*		AGE		Full IQ		ADHD Prevalence		ADOS_TOT_CS
	ASD	TD	ASD	TD	ASD	TD	ASD	TD	ASD
Adults (*N* = 61)	35 M:25 F:10	29 M:21 F:8	29 ± 6.9 M:28.5 ± 7.1 F:30.4 ± 5.7	29.3 ± 6.7 M: 29.1 ± 6.9 F: 30.2 ± 5.4)	109.1 ± 16 M: 107.5 ± 16.9 F: 113.3 ± 14.1	106.6 ± 13.8 M: 109.1 ± 2.6 F: 97.2 ± 12.2	5 (4 M, 1 F) 15%	0	11.1 ± 2.4
Teenagers (*N* = 32)	29 M:24 F:5	11 M:7 F:4	14.1 ± 1.3 M: 14 ± 1.2 F: 15 ± 1	13.9 ± 1.2 M: 14.2 ± 1.1 F: 13.5 ± 1.3	103.1 ± 13.6 M: 101 ± 12.6 F: 126 ± 4	108.6 ± 11.7 M: 111.8 ± 9.4 F: 104.5 ± 13	11 (11 M, 0 F) 48%	0	12.1 ± 0.5
Children (*N* = 36)	33 M:31 F:2	9 M:6 F:3	8.8 ± 1.4 M: 8.8 ± 1.4 F: 8.5 ± 1.5	7.9 ± 1.53 M: 7.3 ± 1.1 F: 8.6 ± 1.7	96.7 ± 15 M: 97.9 ± 15.2 F: 83 ± 6	103.8 ± 14 M: 104.5 ± 16.3 F: 103 ± 11.2	13 (12 M, 1 F) 52%	0	11.7 ± 0.7
Total (All) (*N* = 129)	97 M: 80 F: 17	49 M: 34 F: 11	18.6 ± 8.6 M: 17.5 ± 8 F: 24.9 ± 9.4	21.2 ± 9.55 M: 22.5 ± 9.1 F: 18.5 ± 9.3	103.6 ± 15.9 M: 102.3 ± 15.6 F: 110.6 ± 16.2	106.3 ± 13.4 M: 108.8 ± 12.7 F: 101.1 ± 13	29 (27 M, 2 F) 36%	0	11.6 ± 1.5

Mean values ± SD

IQ, Intelligence Quotient; ASD, Autism Spectrum disorders; F, Females; M, Males; TD, Typical Development; and ADHD, Attention Deficit with or without Hyperactivity Disorders

### Clinical measures

ASD participants were subjected to the DSM-5 criteria evaluation [1] and were rated with standardized diagnostic tools, including the French version of the Autism Diagnostic Interview-Revised [ADI-R, 59] and the Autism Diagnosis Observational Schedule [ADOS, 60]. ASD participants were considered as high functioning, and they were included in the study when their total IQ score was > 70, as assessed with the Wechsler Adult Intelligence Scale (WAIS-III or WAIS-IV) or Wechsler Child Intelligence Scale (WISC IV) [61, 62]. ASD and TD participant executive functions were evaluated with the Behavior Rating Inventory of Executive Function [BRIEF, 63]. The BRIEF clinical scales (Table 3) measure the extent to which the respondent reports problems with different types of behavior related to the nine domains of executive functioning. It consists of equivalent Self-Report (for adults) and Informant Report Forms (for children), each having 75 items in nine non-overlapping scales, as well as two summary “index scales” (Behavioral Regulation Index (BRI) and Metacognition Index (MI)) and a scale reflecting “overall functioning” (Global Executive Composite (GEC)). The Global Executive Composite (GEC) score incorporates all the BRIEF clinical scales, and although useful as an overall summary measure, 2 index subscores (BRI and MI) are especially relevant to establishing an individual’s profile. The Behavior Regulation Index (BRI) captures the person’s ability to regulate and monitor behavior effectively and to inhibit a prepotent response. It is composed of the Inhibit subscore (BRIEF-score1), which is particularly useful for measuring impulsivity, and 3 Self-Monitor (flexibility, motor and emotion monitoring) subscores. The Metacognition Index (MI) is composed of five subscores—Initiate, Working Memory, Plan/Organize, Task Monitor and Organization of Materials—and captures the ability to sustain working memory, plan and organize problem-solving approaches and attend to task-oriented output. Higher scores reflect a higher level of dysfunction (Table 2).

**Table 2:** Oculomotor variables description

Dependent variable name	Description
Latency (ms)	The delay between the onset of the peripheral target and the first non-anticipatory fixation. This variable is used to asses Gap and Overlap effects
Anticipatory saccade (%)	Percentage of trials where saccadic movements occurred within 100 ms following target presentation
Erroneous saccade (%)	Percentage of trials where the first non-anticipatory saccade (> 100 ms) was in the wrong direction as requested by the instructions
Gain to first fixation	The ratio between the actual distance travelled during the first saccade and the theoretical distance required to reach the target

Attention deficits and hyperactivity comorbidity were assessed with the Attention-Deficit/Hyperactivity Disorder Rating Scale (ADHD-RS IV), a questionnaire focusing on the behavior of 4- to 18-year-old children and adults. The ADHD Rating Scale-IV was completed independently by the parents of children or adult participants themselves and scored by a clinician. The scale consists of 2 subscales: inattention, IA (9 items), and hyperactivity–impulsivity, HI (9 items), leading to 2 different subscores. To obtain the total raw score, clinicians add the IA and Hi subscales scores. Higher scores reflect a greater incidence of attention deficits and hyperactivity symptoms. Comorbidity with ADHD was also evaluated using standardized investigative diagnosis tools (Table 3). For the children and teenagers groups, data were collected using the Kiddie Schedule for Affective Disorders and Schizophrenia [K-SADS; 64]. For the adults group, comorbidity with ADHD was assessed using the Diagnostic Interview for Genetics Studies [DIGS, 57]. Table 1 presents the distribution of ADHD indices across the studied population.

**Table 3:** ADHD-RS and BRIEF subscores

	ALL TD	ALL ASD	CHILDREN		TEENAGERS		ADULTS	
			TD	ASD	TD	ASD	TD	ASD
ADHD-RS_Total SCORE	6.90 ± 1.01	23.47 ± 1.45	8.29 ± 1.97	31.33 ± 2.67	6.80 ± 1.52	20.48 ± 2.41	6.13 ± 1.57	20.11 ± 1.99
ADHD-RS-SCORE1 (Inattention Score)	3.6 ± 0.65	15.01 ± 0.96	3.57 ± 1.02	19.10 ± 1.7	5.00 ± 1.36	14.72 ± 1.72	3.04 ± 1.0112	12.21 ± 1.37
ADHD-RS-SCORE2 (Hyperactivity Score)	3.29 ± 0.63	9.8 ± 0.84	4.71 ± 1.58	14.43 ± 1.81	1.80 ± 0.63	7.76 ± 1.25	3.93 ± 0.81	8.14 ± 1.07
BRIEF-GEC (Total Score)	86.8 ± 2.68	145.35 ± 3.67	99.00 ± 6.36	162.05 ± 4.51	91.11 ± 5.51	143.25 ± 7.14	77.67 ± 1.76	134.77 ± 5.84
BRIEF-BRI (Behavioral Regulation Index)	33.71 ± 0.88	56.89 ± 1.64	36.93 ± 2.48	59 ± 2.45	33.2 ± 1.52	53.3 ± 3.16	32.04 ± 0.58	5812 ± 2.76
BRIEF-MI (Metacognition Index)	52.79 ± 1.96	88.46 ± 2.54	62.07 ± 4.2	103.05 ± 2.96	57.44 ± 4.4	89.95 ± 4.49	45.63 ± 1.37	76.65 ± 3.59

Mean values ± sem

### Procedure, task and variable definition

TD participants were rated uniquely at the study’s inception (i.e., time point T0), whereas ASD participants were followed-up and reassessed at 12 months (Y1) and 24 months (Y2) post-T0. Experiments were conducted in a dimly illuminated room in which participants were seated at a distance of 60 cm in front of a Tobii Pro TX300 (Tobii Technology, Stockholm, Sweden), a 23inch screen-based eye-tracker with a sampling rate of 300 Hz. The calibration procedure was carried out before each task presentation, and the head position was maintained fixed on a headrest. Stimuli were displayed on the screen, which had a 1920 × 1080 resolution. Written instructions were presented on screen to participants and were also read aloud by the experimenter. At the beginning of each task (Fig. 1), a central fixation point represented by a green square (0.5 × 0.5 cm) was displayed against a black background. The presentation duration of this anchor point varied from 2000 to 3500 ms. When this initial fixation period had elapsed, a peripheral target represented by a red square (0.5 × 0.5 cm) was displayed for 1000 ms at a distance of 24 cm either on the right or left side of the center point, and participants were requested to switch their gaze to this peripheral target as quickly as possible. In the Gap task, in contrast to the instantaneous transition occurring in the Step task, there was a 200 ms interval (i.e., “Gap”) between the disappearance of the central anchor point and the appearance of the peripheral target. The disappearance of the central point frees activity of the visual fixation system and enables passive attentional disengagement by the participant and, by signaling the imminent appearance of the peripheral target, permits the saccadic system to respond more rapidly to new stimuli [16]. The “Gap effect” is represented by the shorter latencies recorded in the Gap task [27] compared to the Step, and as such, saccade initiation and flexibility provide measures of attention switching ability [26, 65]. In the Overlap task, the peripheral target was displayed as in the Step task, but the central fixation point remained visible on the screen. This task requires a voluntary disengagement of both visual attention and fixation systems to initiate a saccadic displacement from the central anchor point toward the peripheral target [44, 66, 67]; consequently, reaction latencies are longer in the Overlap task compared to those recorded in either the Gap or Step tasks. The deltas between Step latencies and Overlap latencies constitute the so-called Overlap effect, the delta between Gap latencies and Overlap latencies represent the so-called OverGap effect, and we called the delta between Antisaccade and Gap latencies the Antisaccade effect. The Step task is effectively the baseline task, in which saccadic latencies are intermediate-between those in the Gap and Overlap tasks [68, 69]. These 3 different tasks allow the two processes implicated in attention disengagement maturation to be dissociated: automated disengagement assessed by the Gap effect (i.e., Gap–Step latency comparisons) and a higher order process of active disengagement measured by the Overlap and OverGap effects [via Overlap–Step and Overlap–Gap latency comparisons; 40], with the OverGap effect emphasizing both Gap and Overlap effects. Since in Gap, Step and Overlap tasks, participants had to generate saccades toward the target, these 3 tasks constitute the prosaccade task group.

The stimulus presentations in the Antisaccade and Gap tasks were similar, but the task instructions were different. In the Antisaccade task, participants were asked to initiate a saccadic displacement of same magnitude as in prosaccade tasks, but in the opposite direction to the target, which requires an inhibition of the prepotent automatic saccade [named prosaccade; 5, 70, 71]. The Antisaccade task has a higher cognitive cost, and consequently latencies are longer than in the Gap task [72]. This task is useful for studying voluntary and flexible control of movement and inhibition process [24, 52, 55], since the correct achievement of this task requires a top-down inhibition of the reflexive saccade toward a peripheral target located in the other side [70, 73].

Participants were instructed to move their gaze in the peripheral target direction as fast and as accurately possible (for Gap, Step and Overlap tasks) or in the opposite direction to the target (for the Antisaccade task) without any head or body movement. For all four tasks, an initial 4 trial training presentation was provided in order to verify that the participants had fully understood the instructions. Each task consisted of a test block that included 15 trials and lasted for about 90 s for a total session duration of about 20 min. The order of presentation of the four tasks was randomized between participants and time points (T0, Y1, Y2) of the study.

### Data processing and analysis

Data were preprocessed using TobiiStudio™ version 3.3.2 (Tobii Technology, Stockholm, Sweden). For each task, areas of interest (AOIs) were defined around each target so as to detect gaze fixations within a given specific region. For this purpose, a square of 1.4 × 1.4 cm was drawn around the centers of the central fixation point and the peripheral target. Additionally, the screen was divided vertically into two different AOIs of the same dimension (25.1 × 14.5 cm) corresponding to screen-right and screen-left AOIs.

The parameters of ocular fixations (*X* and *Y* positions in pixels, latency values, accuracy, inside/outside AOIs) and time stamps of the central fixation point and peripheral target presentations were exported from TobiiStudio™. A custom-built toolbox developed with MATLAB version 2020a (The MathWorks, Inc., Natick, MA, USA) was used to compute the dependent variables presented in Table 2. First, our algorithm automatically checked for data quality and rejected eye tracking data which displayed a poor validity index, i.e., according to Tobi preprocessing were > 2 on the Tobi index that ranges from 0 (excellent) to 4 (no eye detected). Trials in which the subject was not looking at the fixation point (tolerance of 4 cm) when the target appeared were also rejected. Second, we identified anticipatory and erroneous saccades. The anticipatory saccade percentage was the percentage of trials in which premature saccades (i.e., saccades occurring < 100 ms following target appearance) occurred. The erroneous saccade percentage was the percentage of responses where the first non-anticipatory saccade was in the wrong direction (I.e., toward rather than away from the target). Third, for non-anticipatory and non-erroneous saccades, we computed response time and gain. The response time parameter that we used was the latency to first fixation which included the reaction time (i.e., the time that elapsed between the appearance of the target and the onset of a saccade in response to that target) plus the saccade duration until a first fixation occurred. The gain to first fixation allowed measurement of saccade accuracy, i.e., how precisely the saccade directs the eye to the target, computed as the ratio of the actual distance travelled during the first saccade over the theoretical distance required to reach the target. For each trial, latency and gain were considered only if the first saccade had travelled a third of the required distance to reach the target.

Statistical analysis was performed on the dependent variables using MATLAB 2021a. We used the “outlier” function in MATLAB, which removes elements that exceed a 3-scaled median absolute deviation (MAD) away from the median. Outliers, erroneous and anticipatory saccades were not included in the latency and gain calculations. All data passed normality (Kolmogorov–Smirnov test). All the variables and delta measures were analyzed separately using repeated measures ANOVAs. When main effect or interaction was significant, we reported the effect size using Eta-squared (*η*^2^), that is a descriptive measure of the strength of association between independent and dependent variables in the sample. A first analysis was conducted on the data obtained at T0. Attentional performances were compared in a mixed-design ANOVA with Task (Gap/Step/Overlap) as a within-subject factor and Group (ASD/TD) and age group (children/teenagers/adults) as two between-subject factors. Inhibitory control performances were compared in another mixed-design ANOVA with Task (Gap/Antisaccade) as a within-subject factor and group (ASD/TD) and age group (children/teenagers/adults) as two between-subject factors. Trial-to-trial variability in task performance was also tested at T0 using the coefficient of variation (COV; ratio of the standard deviation to the absolute value of the mean) for latencies and gain. Mixed-design ANOVA was conducted on COV with the same factors used for performance averages.

The three time-point analysis (T0-Y1-Y2) for the ASD group (34 adults, 29 teenagers, 29 children) was first performed using a mixed-design ANOVA with Task (Gap/Step/Overlap) and Time Point (T0-Y1-Y2) serving as a within-subject factor and age group (children, teenagers, adults) as between-subject factors. A second mixed-design ANOVA was performed with the Gap/Antisaccade tasks and Time Point as within-subject factors, and with age group as a between-subject factor. For each comparison, when an age group effect was observed without any differences between the ASD and TD groups, we will use the terms “All Children,” “All Teenagers” and “All Adults.” The level of significance was set at *p* = 0.05. Post hoc tests were performed with a Tukey–Kramer correction to adjust for multiple comparisons.

To test whether oculomotor responses were linked to clinical atypicalities in executive and attentional functions, we conducted Pearson bivariate correlations between the oculomotor variables in our T0 data set and the ADHD-RS and BRIEF questionnaire scores. Attentional function scores were thus used as an outcome to be correlated with the oculomotor functions. As such, the ADHD-RS total score was a controlling variable for computing the partial correlation between BRIEF and oculomotor variable. To investigate the link between clinical atypicalities in executive functions on the capacity for visual disengagement and inhibition, the deltas between latencies and the erroneous and anticipatory saccade percentages were correlated with the questionnaire scores. The p value was adjusted for multiple correlations using a Holm–Bonferroni sequential correction [74]. A Chi-squared test was performed to compare the ADHD comorbidity rating between children, teenagers and adults in the ASD and TD cohorts. In the text, values for all variables are expressed as means ± standard error of the mean (sem), unless specified.

## Results

Our findings are successively presented for each dependent variable (Table 2) in the following three main sections: (1) comparisons between the different ASD and TD participant groups at T0; (2) correlations between the results of oculomotor tasks and clinical data; and (3) the evolution of ASD participant measurements through the following 2 years.

### Comparative analysis of visually guided saccades in oculomotor tasks at T0

The data collected for the 4 selected oculomotor variables (Table 2) are presented in Additional file 1. For each dependent variable, we first conducted a three-way ANOVA (Clinical group × Age group × Task) to establish whether a main effect and interaction occurred.

#### Visual attention and disengagement

##### Latencies

**Fig. 2:**
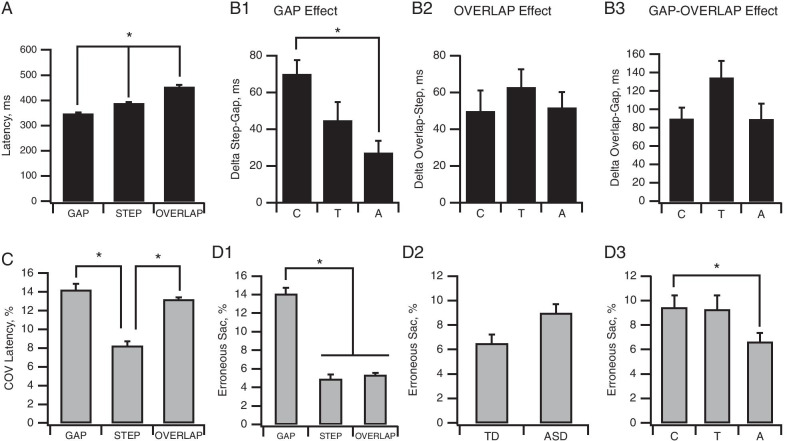
Gap and Overlap task effects. **A** Plots of mean latency values in the three tasks (All participants, *N* = 146). Note the progressive significant increase in latency from Gap to Overlap task. **B** Gap (**B1**) and Overlap (**B2**) effects assessed by computing the differences in latency between Step and Gap tasks and between Overlap and Step tasks. **A** Adults (*N* = 64); **C** children (*N* = 42); T: teenagers (*N* = 40). **C** Plots of mean coefficient of variation (COV) of latency values in the three tasks (All participants, *N* = 146). Bars indicate the standard error of the mean; *p < 0.05

We performed an analysis on the absolute value of latencies recorded in the various tasks to first establish the overall effect of each task on latency values (Fig. 2A). Subsequent analyses were performed on delta measures. A three-way ANOVA (Clinical group × Age group × Task) performed on latency measurements in the Gap, Step and Overlap tasks revealed a main effect of the task (*F*(2,190) = 83.6, *p* < 0.001, *η*^2^ = 0.27). Post hoc analysis showed significant differences (all *p* < 0.001) between Gap, Step and Overlap latencies, which had mean values of 348.16 ± 4.97 ms, 389 ± 3.9 ms and 453 ± 7.72 ms, respectively (Fig. 2A).

We also found a significant main effect of age group (*F*(2,103) = 5.9, *p* = 0.004, *η*^2^ = 0.1) at T0, for the Gap effect (Fig. 2B1), which was significantly stronger for all children than for all adults (70.12 ± 7.42 ms vs. 27.2 ± 6.42 ms, *p* = 0.004), whereas the teenager group (48.11 ± 7.42 ms) was not significantly different from the other two groups. No significant clinical group effect was observed for the Gap effect (*F*(1,103) = 0.65, *p* = 0.42). The Overlap effect (Fig. 2B2) was not significantly affected by clinical group (*F*(1,100) = 1.21, *p* = 0.27), nor by the age group (*F*(2,100) = 0.92, *p* = 0.40), and the interaction was not significant (*F*(2,100) = 1.24, *p* = 0.29). The delta Overlap–Gap (Fig. 2B3) was significantly affected by a clinical group × age group interaction (*F*(2,108) = 3.7, *p* = 0.028, *η*^2^ = 0.06). Post hoc analysis revealed a significant difference between TD and ASD for teenagers only (TD: 172.4 ± 25.4 ms, ASD: 60.4 ± 18.3 ms, *p* = 0.015). Data collected for the delta values are provided in Additional file 2.

Analysis of the coefficient of variation (COV) of latencies to first fixation (Fig. 2C) revealed a significant Task effect (*F*(2,142) = 14.36, *p* < 0.001, *η*^2^ = 0.1), with a significantly greater (*p* < 0.001) trial-to-trial variability occurring in the Gap (14.25 ± 0.75%) and Overlap tasks (13.21 ± 0.71%) than in the Step task (8.2 ± 0.32%). No effect of age group (*F*(2,71) = 1.19, *p* = 0.31), clinical group (*F*(1,71) = 0.79, *p* = 0.38) or any significant interaction was observed.

##### Erroneous saccade percentages

Three-way ANOVA revealed a main effect of task (Fig. 2D1; *F*(2,220) = 34, *p* < 0.001, *η*^2^ = 0.15), with a higher percentage of erroneous saccades occurring in the Gap task (14.1 ± 1.07%) compared to both the Step (4.92 ± 0.68%) and Overlap tasks (5.35 ± 0.73%). A main effect of age group was also evident (Fig. 2D3; *F*(2,110) = 3.08, *p* = 0.05, *η*^2^ = 0.05) with higher erroneous saccades performed by children compared to adults. There was no significant main effect of clinical group (Fig. 2D2; *F*(1,110) = 1.97, *p* = 0.16).

##### Anticipatory saccade percentages

**Fig. 3:**
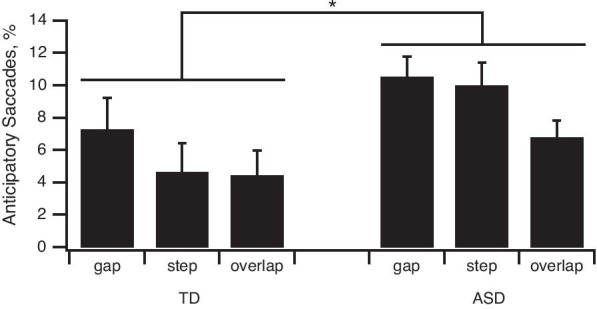
Anticipatory saccade. Plots of mean percentage of anticipatory saccades performed in the three tasks. ASD participants made significantly more anticipatory saccades than their TD peers in the three tasks. Bars indicate the standard error of the mean; *p < 0.05

Three-way ANOVA revealed a significant main effect of clinical group (Fig. 3; *F*(1,110) = 5.17, *p* = 0.025, *η*^2^ = 0.04) for this variable. ASD participants made more anticipatory saccades than their TD counterparts at T0 (ASD: 9.1 ± 0.74%; TD: 5.47 ± 1%). A main effect of task was also nearly significant (*F*(2,220) = 2.94, *p* = 0.055, *η*^2^ = 0.01), with a higher percentage of anticipatory saccades occurring in the Gap task (9.33 ± 1.06%) compared to both the Step (8.12 ± 1.14%) and Overlap tasks (5.91 ± 0.87%).

##### Gain

**Fig. 4:**
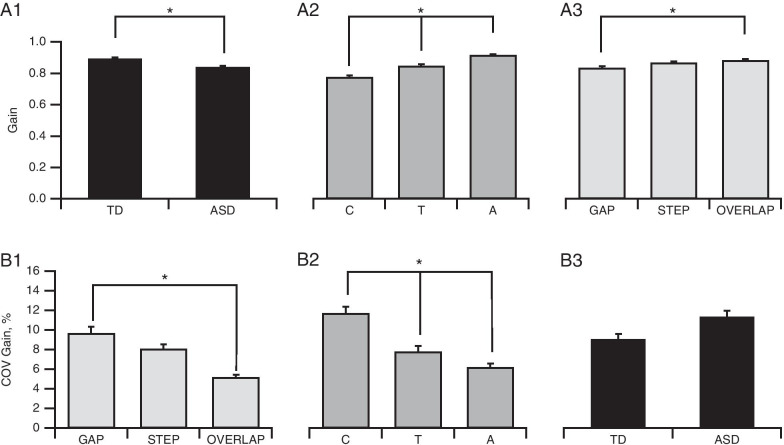
Analysis of accuracy in Gap, Step and Overlap tasks. **A** Plots of mean gain values. There was a significant difference in gain to first fixation between ASD and TD participants (**A1**), in the course of development (**A2**) and between tasks (**A3**). **B** Plots of mean coefficient of variation of gain values. While there were no significant differences between ASD and TD participants, there was a significant developmental improvement between age groups (**B2**), and between tasks. Bars indicate the standard error of the mean; *p < 0.05

Three-way ANOVA revealed a significant main effect for clinical group (Fig. 4A1; *F*(1,96) = 4.22, *p* = 0.043, *η*^2^ = 0.03), with a higher gain found for All TD (0.9 ± 0.01) than All ASD participants (0.84 ± 0.01). A significant main effect of age group (Fig. 4A2; *F*(2,96) = 25.76, *p* < 0.001, *η*^2^ = 0.34) was also observed, with significant differences of gain occurring between all three groups: adults (0.92 ± 0.01), teenagers (0.85 ± 0.01) and children (0.77 ± 0.01; all *p* < 0.01). A main effect of Task (Fig. 4A3; *F*(2,192) = 6.6, *p* < 0.002, *η*^2^ = 0.03) was also evident, and post hoc analysis only revealed a significantly (*p* < 0.001) higher gain in Gap (0.84 ± 0.01) than in Overlap (0.88 ± 0.008) tasks.

A significant main effect of Task was present for trial-to-trial variability (Fig. 4B1; *F*(2,98) = 8.1, *p* < 0.001, *η*^2^ = 0.08), with a significantly higher trial-to-trial variability occurring in the Gap (9.7 ± 0.61%, *p* = 0.001) and Step (8.08 ± 0.48%, *p* < 0.001) tasks than in the Overlap task (5.2 ± 0.23%). A main effect of age group was also evident (Fig. 4B2; *F*(2,49) = 7.36, *p* = 0.002, *η*^2^ = 0.23). Children had a higher COV in gain to first fixation (11.73 ± 0.66%) compared to adults (6.24 ± 0.35%, *p* = 0.001) and teenagers (7.8 ± 0.56%, *p* = 0.053 only). No effect of clinical group (Fig. 4A; *F*(1,49) = 0.009, *p* = 0.93) nor any interaction was observed.

#### Inhibition

##### Latencies

**Fig. 5:**
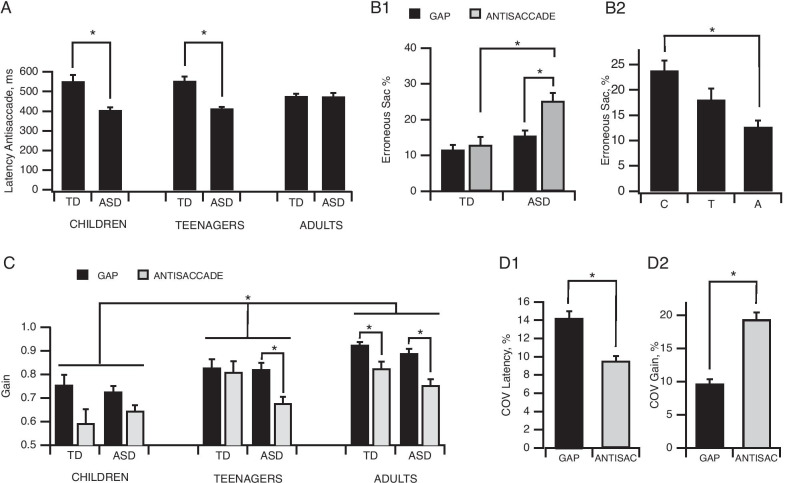
Antisaccade task effects. **A** Plots of mean differences in latency between Gap and Antisaccade tasks indicating significant differences between clinical groups for children and teenagers. **B** Plots of mean gain values indicate significant differences between age groups and significant differences in accuracy in the Gap task versus Antisaccade task for ASD participants. **C** ASD participants performed more erroneous saccades in the Antisaccade task compared to Gap task and more than TD participants in the Antisaccade task (**C1**). The percentage of erroneous saccades decreased over development (**C2**). **D** Plots of mean coefficient of variation of latency (**D1**)/gain (**D2**) indicating significant differences between the two tasks. Bars indicate the standard error of the mean; *p < 0.05

Three-way ANOVA of latencies revealed a significant main effect of the Antisaccade task for all participant groups, whatever the age or clinical category. A significant age group × clinical group × task (Gap vs. Antisaccade) interaction was present (*F*(2,81) = 7.7, *p* < 0.001, *η*^2 =^ 0.034). Post hoc analysis indicated that latencies (Fig. 5A) were higher in the Antisaccade task for TD than ASD in children and teenagers (children: TD 551.57 ± 34 ms, ASD 405.18 ± 15.6, *p* = 0.02; teenagers: TD 553.49 ± 22.92, ASD 414.3 ± 39.8, *p* < 0.001), although no significant difference was evident between adults (TD: 477.34 ± 11.95; ASD: 474.84 ± 17.55 ms; *p* = 0.85).

ANOVA performed on the COV of latencies (Fig. 5D1) showed a task effect (*F*(1,55) = 7.76, *p* = 0.007, *η*^2^ = 0.08), with post hoc comparisons revealing a higher trial-to-trial variability in latencies for prosaccades (GAP task) than for antisaccades (14.25 ± 0.75% vs. 9.57 ± 0.51%).

##### Erroneous saccade percentage

Three-way ANOVA of erroneous saccades indicated a significant clinical group × task interaction (*F*(1,108) = 5.09, *p* = 0.026, *η*^2^ = 0.02). Post hoc comparisons showed that only ASD participants made a significantly higher percentage of erroneous saccades in the Antisaccade task compared to the Gap task (Fig. 5B1; 20.89 ± 1.7% vs. 14.08 ± 1.07%, *p* < 0.001). Post hoc comparisons showed that ASD participants made a significantly higher percentage of erroneous saccades than TD participants (Fig. 5B1; 25.22 ± 2.22% vs. 12.94 ± 2.26%, *p* = 0.012). A main effect of age group was also observed (Fig. 5B2; *F*(2,108) = 6.74, *p* = 0.002, *η*^2^ = 0.11), with a significantly higher percentage of erroneous saccades for children (23.31 ± 2.45%) than for adults (12.73 ± 1.56%, *p* < 0.001). Teenagers (19.1 ± 2.23%) did not express erroneous saccade percentages that were significantly differed from children (*p* = 0.22) or adults (*p* = 0.35).

##### Gain

Three-way ANOVA conducted on gain values in the Antisaccade task indicated a significant interaction between clinical group × age group × task (Gap vs. Antisaccade; *F*(2,92) = 3.26, *p* = 0.042, *η*^2^ = 0.03). Post hoc comparisons showed that the gain was significantly higher in the Gap task than in the Antisaccade task (Fig. 5C) for TD adults (0.92 ± 0.02 vs 0.83 ± 0.02, *p* < 0.001), ASD adults (0.89 ± 0.02 vs 0.75 ± 0.02, *p* < 0.001) and ASD teenagers (0.82 ± 0.02 vs 0.68 ± 0.03, *p* = 0.003), but not for TD teenagers (0.83 ± 0.04 vs 0.81 ± 0.05, *p* = 0.57), ASD children (0.73 ± 0.02 vs 0.65 ± 0.02, *p* = 0.26) and TD children (0.76 ± 0.04 vs 0.59 ± 0.06, *p* = 0.08).

Three-way ANOVA on the COV of gain values revealed a main effect of task (Fig. 5D2; *F*(1,61) = 16.26, *p* < 0.001, *η*^2^ = 0.11), the COV being significantly greater in the Antisaccade task than in the Gap task (19.38 ± 1.06% vs. 9.7 ± 0.64%, respectively).

### Correlations between oculomotor functions and clinical variables

We next used correlation analysis to examine possible relationships between our oculomotor variable measurements and the rating scales for ADHD comorbidity and attentional functions (ADHD-RS total scores plus the two subscores) and for executive functions (BRIEF-GEC and BRIEF-BRI scales, with a higher score reflecting the level of impulsivity (see Material and Methods for details on clinical scales)). In particular, we were interested in correlations that could provide explanatory insights into the results presented above.

#### ADHD-rating scale

Comorbid ADHD was found only in ASD participants, affecting 36% of the group (*Χ*^2^(1) = 21.34, *p* < 0.001), and was distributed as follows: 52% ASD children (*Χ*^2^(1) = 9, *p* = 0.003), 48% ASD teenagers (*Χ*^2^(1) = 6.5, *p* = 0.01) and 15% ASD adults (*Χ*^2^(1) = 4.3, *p* = 0.03). We examined correlations between the delta Overlap–Gap and ADHD scores (total score and both subscores) since the Overlap tasks require the highest levels of attentional and executive control. Similarly, since erroneous and anticipatory saccade percentages indicate possible global attentional and inhibition dysfunctions, we tested correlations between these variables and ADHD scores.

**Fig. 6:**
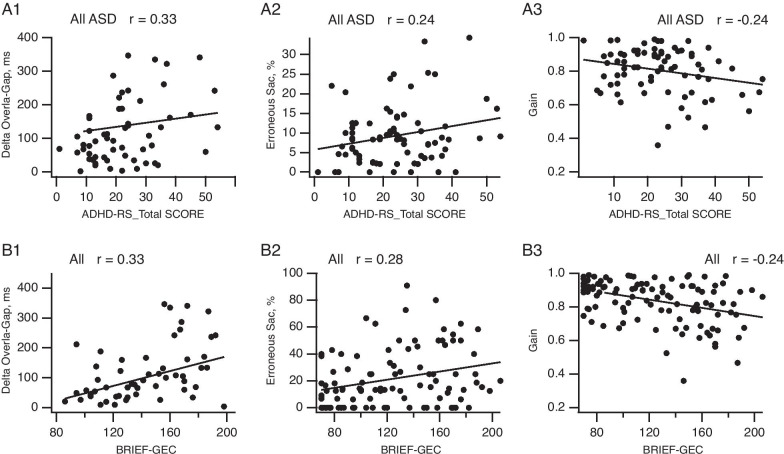
Correlations between oculomotor variables and clinical measures. **A** Correlation plots were drawn for ASD participants between ADHD-RS total score and delta Overlap–Gap, erroneous saccade percentage and gain (averaged values from all trials in prosaccade tasks. **B** Correlation plots were drawn for all participants between BRIEF-GEC and delta Overlap–Gap, erroneous saccade percentage and gain (averaged values from all trials in prosaccade tasks). Bars indicate the standard error of the mean; *p < 0.05

In all ASD participants, the delta Overlap–Gap (Fig. 6A1) was positively correlated with the total ADHD-RS score and both subscores (total *r* = 0.33, *p* = 0.021; score 1: *r* = 0.27, *p* = 0.036; score 2: *r* = 0.33, *p* = 0.036). Moreover, erroneous saccade percentages (Fig. 6A2) in all prosaccades tasks were positively correlated with the ADHD total score (*r* = 0.24, *p* = 0.046; score 2: *r* = 0.26, *p* = 0.046). On the other hand, the gain (Fig. 6A3) was negatively correlated with the total score of the ADHD-RS (*r* = -0.024, *p* = 0.036).

#### BRIEF scale

Again, as the Overlap tasks and Antisaccade tasks required the highest levels of attentional and executive controls, we tested correlations between the delta Overlap–Gap and both the total GEC scores and the BRI subscores of the BRIEF scale. We also tested the correlation between the erroneous saccade percentage in the prosaccade tasks and in Antisaccade task with the 2 BRIEF scores. Additionally, we examined correlations between anticipatory saccade percentage in the Gap task and clinical measures of impulsivity though BRIEF-BRI subscore.

For All participants, the total BRIEF-GEC score (Fig. 6B1) and the BRI subscore were positively correlated with the delta Overlap–Gap (BRIEF-GEC: *r* = 0.22, *p* = 0.04; BRIEF-BRI: *r* = 0.19, *p* = 0.05). BRIEF-GEC score and the BRI subscore were also significantly correlated with anticipatory saccades percentages in prosaccade tasks (BRIEF-GEC: *r* = 0.2, *p* = 0.05; BRIEF-BRI: *r* = 0.21, *p* = 0.05), while erroneous saccades percentages in Antisaccades task were only significantly correlated with the BRIEF-GEC (Fig. 6B2; BRIEF-GEC: *r* = 0.28, *p* = 0.01, BRIEF-BRI: *r* = 0.18 *p* = 0.07). For all ASD participants, the correlation with the delta Overlap–Gap was significantly positive and remained significant after controlling for ADHD index (partial correlation with BRIEF-GEC: *r* = 0.26, *p* = 0.05; BRIEF-BRI: *r* = 0.16 *p* = 0.25). For both TD and ASD participants, the BRIEF-BRI subscores were positively correlated with latencies in the Antisaccade task (TD BRIEF-BRI: *r* = 0.43, *p* = 0.01, ASD BRIEF-BRI: *r* = 0.30, *p* = 0.056).

For all participants, the BRIEF-GEC was negatively correlated with the gain in prosaccades tasks (Fig. 6B3; partial correlation with ADHD-RS as controlling variable, BRIEF-GEC: *r* = -0.23, *p* = 0.036; BRIEF-BR: *r* = -0.05, *p* = 0.62). This correlation did not remained significant for ASD participants after controlling for ADHD index (partial correlation with ADHD-RS as controlling variable, BRIEF-GEC: *r* = -0.16, *p* = 0.19; BRIEF-BRI: *r* = -0.05, *p* = 0.68). In the Antisaccade task, the BRIEF-GEC was also negatively correlated with the gain for ASD (BRIEF-GEC: *r* = -0.28, *p* = 0.04) and TD participants (BRIEF-GEC: *r* = -0.35, *p* = 0.04).

#### Stability/variability of oculomotor performance in ASD participants over time

**Fig. 7:**
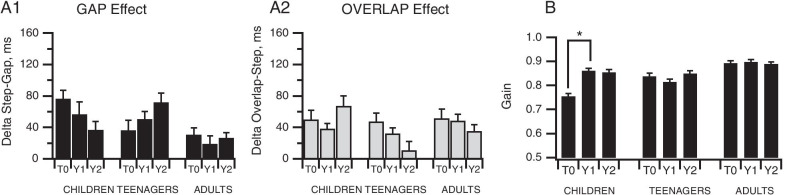
Analysis of changes in oculomotor variables over 2 years of follow-up. **A** Plots of mean latency difference values to assess changes in Gap and Overlap effects over 2 years. **B** Plots of mean gain values over 2 years. T0: Study onset; Y1: year 1; Y2: year 2. Bars indicate the standard error of the mean; *p < 0.05

The mixed design ANOVAS (see Methods) at 3 time points T0-Y1-Y2 from our 2-year follow-up evaluation of ASD participants revealed a maintenance of the main effects of task observed at T0 in All ASD participants for Latencies between Gap, Step and Overlap tasks (*F*(2,22) = 13.22, *p* < 0.001) and between the Gap and Antisaccade tasks (*F*(1,13) = 27.75, *p* < 0.001). However, analysis of the Gap effect (Fig. 7A1) over the 2-year study for ASD participants did not reveal any significant effect of age group (*F*(2,19) = 1.14, *p* = 0.33), of year (*F*(2,38) = 0.035, *p* = 0.97), and no age group × year interaction (*F*(4,38) = 1.11, *p* = 0.37). Similarly, age group (*F*(2,19) = 0.35, *p* = 0.71) and year (*F*(2,38) = 1.98, *p* = 0.15) did not significantly alter the Overlap effect (Fig. 6A2), again without any interaction between these two parameters (*F*(4,38) = 0.97, *p* = 0.44).

In Step/Gap/Overlap task comparisons over the two-year period, we found an effect of time point in the children concerning gain (Fig. 7B; *F*(4,32) = 5.553, *p* = 0.002). Children exhibited a significantly higher gain (0.86 ± 0.01) at Y1 compared to T0 (0.75 ± 0.01, *p* < 0.001). However, no effect of year either on erroneous or anticipatory saccade percentages was observed. Data collected for the delta values at the three time points are provided in Additional file 3.

## Discussion

The main goal of this study was to identify and compare differences in spatial attention and inhibitory control in ASD and TD participants by means of different oculomotor tasks performed at three developmental stages (children, teenagers, adults) and at three successive time points (T0-Y1-Y2) for the ASD participants, in a 2-year follow-up evaluation. The implications of ADHD comorbidity and executive function skills correlations were also considered.

## Summary of findings

First, a significant task-dependent effect was detected, confirming the requirement for a progressive increase in attention and inhibition through the Gap, Step and Overlap tasks until ultimately, the Antisaccade task. Task comparisons among all participants revealed distinct Gap, Overlap and an effect of the Antisaccade task with a progressive increase in latencies. Participants displayed greater trial-to-trial variability in their prosaccade latencies and gain in the Gap and Overlap tasks, compared to the Step task and in Antisaccade tasks compared to Gap task (Figs. 2C, 5D1). They also displayed more erroneous and anticipatory saccades in the Gap task compared to all other tasks (Figs. 2D1, 5B1).

The task effect and intra-individual (trial-to-trial variability) differences in latencies between these tasks have already been reported in a TD subjects population [75, 76]. Trial-to-trial differences are likely to be due to fluctuations in attention and corresponding changes in brain activity during a task performance. Saccade movement in the Gap task has been proposed to be controlled by both reflexive (low-level processes of ocular fixation) and volitional commands, requiring cognitive and visual control to maintain central fixation during the gap period, when no fixation target is present, in contrast to the Step or Overlap tasks. In an fMRI study, Ozyurt et al. [75] speculated that higher-level processes (in the fronto-parietal regions) that serve to prevent premature saccades in the Gap task could be engaged to a different degree between subjects and between trials and differ from those regions engaged in other paradigms, such as the Overlap task. This involves the recruitment of fixation neurons that have been identified in frontal eye fields (FEFs) and the lateral intraparietal area (LIP), whose activity is modulated after the disappearance of a foveal fixation point during active fixation in the gap interval. The shortening (Gap effect), but also the high intra-individual variability of saccade latencies in the Gap paradigm, has been attributed to specific but highly variable activations of these frontal regions, although this conclusion must be treated with caution since brain activity in oculomotor-related regions prior to task initiation was not studied. Indeed, trial-to-trial fluctuations in pre-stimulus activity have been shown to predict many domains of behavior and perception [for review, see 77], and it is possible that this type of predictability extends to oculomotor behavior. Moreover, anticipatory saccade rate was correlated with the clinical measure of impulsivity in the cohort examined.

In the overlap task, more effort and volition are required for saccade initiation to overcome the ocular fixation reflex induced by the fixation point when the peripheral saccade target appears. This task induces saccades with a prolonged latency that reflects three operations: attentional engagement with a fixation of gaze toward the fixation point, disengagement from this point and reorientation toward the new stimulus.

The Antisaccade task appears to constitute the most effective oculomotor task for detecting participants with inhibitory control deficits and for determining mature inhibitory function issues, since our measurements with this paradigm indicate the lowest gain and the highest percentage of erroneous saccades. The COV of both latencies and gain was also significantly higher in this task compared with the Gap task, suggesting that the Antisaccade task best demonstrates the capability of consistent inhibition control, in accordance with previous data [78].

Second, a typical developmental effect was observed, with a progressive enhancement of attention disengagement and inhibition skills occurring with age. This was evidenced by: (1) a stronger GAP effect found in all children compared with all adults (Fig. 2B1), an improvement in performance (Fig. 4A2, Fig. 5C) along with an increase in gain in all four tasks, and a decrease in the COV of gain (Fig. 4B2) with age; (2) a reduction in impulsivity with maturation, as indicated by the age-correlated decrease in the percentage of erroneous saccades in the four tasks (Figs. 2D3, 5B2). The reduction in the Gap effect with age indicated an improvement in cognitive control in the transition from childhood to adulthood, suggesting that children may rely more on the protective effect of fixation than mature participants, which is in line with previous results [79]. Moreover, this is consistent with a developmental improvement of executive functions in both typical [24, 31, 80–82] and ASD subjects [34, 55, 78], and in accordance with previous findings [39]. The age when adult-level performance is attained varies between previous studies. A body of evidence has suggested that maturity is reached by age 14–15 years for latency, including in the Antisaccade task, whereas according to other studies, maturity is not reached until the twenties [33, 78, 79] which may be linked to variability in the criteria used. In contrast, the developmental course of attention switching capacities seems to be more complex, since the Gap–Overlap effect increases from childhood to adolescence in non-ASD Teenager group.

Third, from ASD/TD comparisons, a clinical group effect was revealed whereby ASD participants showed (1) less accuracy, since the gain in all prosaccade tasks was significantly smaller in ASD groups and (2) more frequent errors in their oculomotor performances, since ASD groups exhibited more anticipatory saccades in all prosaccade tasks and more erroneous saccades in the Antisaccade task, compared to TD groups (Figs. 3, 4A1, 5B1) as found previously [78, 83], (3) no increase in the Overlap effect from childhood to adolescence.

Fourth, clinical attentional and executive functioning were correlated with oculomotor performance: Specifically, the Gap–Overlap, Antisaccade effects, the gain and both anticipatory and erroneous saccade percentages in all tasks were correlated with ADHD and/or BRIEF total scores.

Fifth, in ASD subjects, there was no improvement in oculomotor behavior over the two-year study period, with the exception of gain in ASD children (Fig. 7).

### Gap and Overlap effects: flexibility and disengagement of attention in ASD

Although there are strong indications from early development studies in infants, that visual disengagement is impaired in ASD and significantly differs from TD peers at 12 months of age in high-risk toddlers who later receive a diagnosis of ASD [84; for review, see 16], other studies on older children, teenagers and adults are more discordant, [22; for review, see 16]. Many studies pointed that orienting and disengagement are impaired in ASD relative to control participants in childhood and adulthood (with lower latencies in the prosaccade tasks and in the overlap task in particular), but with higher group differences when visual tasks used social cues or dynamic and/or color images [16, 84]. Since ASD subjects exhibit atypicalities in processing visual stimuli over a broad spatial region due to a narrow attentional focus [85–87], the use of larger visual stimuli may implicate visual attention issues that could negatively influence ASD participant latencies.

In agreement with previous findings, prosaccade Gap and Overlap effects were observed in the present study in all ASD participants and did not significantly differ from control TD groups. We did not find significant differences in either latencies or the COV of latencies between ASD and non-ASD participants in prosaccades tasks, except in teenager groups. This could further indicate a preservation of typical-like prosaccade effects in ASD participants at least at childhood and adulthood. Regarding the GAP effect, previous studies have suggested that a typical-like flexibility in overt attention switching is preserved in ASD subjects, [5, 24, 35, 40, 53, 65, 88, 89], although some results have reported difficulties in disengaging attention in young ASD participants [16], as also indicated by the absence of an Overlap effect in ASD children [23, 40, 90]. However, this was not supported by other data, particularly in adults, but also in teenagers and children with ASD [24, 91–94].

These apparently contradictory findings could be due not only to the age of the ASD participants studied, but also to differences in methodological approaches, and in particular: (1) the type of stimuli used (using the same static stimuli for the peripheral and central image may not capture attention as easily as a novel or dynamic peripheral stimulus); (2) the inter-stimulus interval (ISI) duration (ASD is slower to disengage for ISI shorter than 500 ms but does not present significant different performances from TD for ISI > 800 ms); (3) the testing methods (studies that separated the Gap and Overlap trials in different blocks may have impacted the Gap and Overlap effects). The delay in disengagement for short ISI may suggest that top-down preparation of the saccadic response is impaired in ASD and requires additional time to shift attention to a peripheral stimulus in the visual field [16, 23, 40, 84, 90].

Furthermore, there is no standard procedure in the way these oculomotor tasks are analyzed. In some studies, the saccadic latencies recorded in the Gap task are compared only to Overlap task measurements (Delta Gap–Overlap, Fig. 1) [23, 35, 40, 81, 88, 89], whereas other studies compared the latencies monitored in the Gap task relative to those in the Step condition [22, 52, 65, 94]. In contrast, the Step–Overlap latency relationship has been less frequently investigated [23, 35, 40, 88, 91, 92, 95].

In line with several studies reported below, our results show no overall significant differences between ASD and TD groups neither in latency values in Gap, Step or Overlap tasks, nor in the computed Gap or Overlap Effects, except between teenager groups. Our results, as a matter of fact, were unexpected for the teenager groups, with a smaller OverGap effect (delta Gap–Overlap) in ASD teenagers. These are, however, in line with Van der Geest et al. [88], Kawakubo et al. [40] and Todd et al. [35], who did not find group differences in adult and teenager samples in pro-saccades latencies values, using basic and static cues in their tasks. Our study design is particularly very similar to the one used by Vander der Geest et al. [88] (static simple stimuli, ISI of 1000 ms, dark background, separate blocks for each tasks) that found no differences between ASD and TD teenager groups (*n* = 16) in latencies values in Gap and Overlap tasks but who, in line with our results, specifically found that the Overlap effect (delta between Overlap latencies and Gap latencies) was smaller in the ASD group than in the TD group.

These findings could illustrate the complex issue of visual disengagement testing already pointed out in the literature above mentioned and may account for the high degree of discrepancy between studies. This effect may also be driven by the very small size of the TD groups (*n* = 9), the high inter-subject variability in latencies measures in ASD group and by the ASD group having very slightly (but not significantly) slower latencies in the Gap task and slightly (but not significantly) faster latencies in the Overlap task compared to the TD group. Taken together, these null and/or unexpected results may then suggest that the real differences between ASD and TD participants found in the literature concerning latency measures might be related to particularities in stimulus processing capacities of ASD individuals and do not necessarily reflect abnormal disengagement of visual attention. However, another explanation can be proposed. In our findings, while the Gap effect decreased with age in both groups, the Overlap effect increased somewhat from children to teens in the TD group but remained more consistent across age groups in the ASD group. This could demonstrate true differences between groups in the development of attention switching compared to that of visual disengagement. This second interpretation should be reinforced by further studies, controlling for methodological issues and focusing on the specific developmental window of adolescence in order to better describe differences in visual disengagement and attention switching in typical and atypical neurodevelopment.

Our results are nevertheless consistent with earlier evidence for the presence of particularities in orienting, shifting and disengaging attention in participants with ASD, although ASD appears to impact more specifically on accuracy and rates of saccade errors, since the gains in all prosaccades tasks were lower and percentages of anticipatory saccades higher in ASD than TD participants [7, 24, 36, 38, 38].

In All ASD participants, the correlations with clinical outcomes indicate that attention disengagement (i.e., the Gap–Overlap effect) is related to higher inattention and inhibition scores as assessed by ADHD-RS and BRIEF scale indices, in accordance with Vaidya et al. [96].

### The Antisaccade task and inhibitory control in ASD

Our data suggest that even if this inhibitory control process emerges progressively through development and improves from childhood to adulthood during both typical and atypical development in ASD [49, 55, 56, 97], it is nonetheless affected by the ASD condition. All ASD participants invariably exhibited higher rates of erroneous saccades than their TD counterparts. Erroneous saccades in the anti-saccade might index a failure to inhibit a reflexive saccade, while in the context of Gap/Step/Overlap they might reflect anticipatory behaviors (a look to one side was planned before target onset). Impulsivity, less precise saccades and additional compensatory saccades to reach peripheral targets have been reported in participants with ASD [7] suggesting abnormalities in cerebellar circuitry and specifically within structures such as the oculomotor vermis (lobules VI–VII), fastigial nuclei and parapontine reticular formation [39].

The performance in the Antisaccade task was not significantly different in ASD and TD individuals in the three developmental stages, as reported previously [55, 78]. However, latencies were significantly higher for TD children and teenagers than their ASD peers, although no latency differences were observed between ASD and TD adults. These results are in accordance with the findings of Minshew et al. [98] who did not find differences between ASD and typical subjects either in adolescents or adult samples, and of Luna et al. [55] in a cross-sectional study that included individuals between 8 and 33 years old. This could be related to impulsivity and/or inhibitory control dysfunctions in ASD, but could be also related to the smaller TD sample size which was smaller in TD and/or by a gender bias (see Limitations section). An impact of ASD on age‐related improvements in inhibitory control during early adolescence was also indicated by our finding of atypical oculomotor behaviors expressed specifically in the adolescent ASD subgroup, in line with IRM data that highlight this important developmental period [24, 39, 46]. A fMRI study using an Antisaccade task suggested that brain circuitry underlying inhibitory control develops differently in ASD. Specifically, there may be relative underdevelopment of brain processes underlying inhibitory control in adolescents with ASD, which may lead to engagement of subcortical compensatory processes [56].

The shape of the developmental trajectory in ASD remains poorly understood, and our data confirmed particularities and inconsistencies in younger developmental stages (childhood and adolescence) [55]. As adulthood is reached in autism, latencies of antisaccades were similar to TD group and performances (accuracy, errors rates) had significantly improved from childhood. These results suggest that some aspects of the abnormal latencies of the Antisaccade task may represent a transitory problem during childhood and adulthood. Taken together with the preserved prosaccade effects reported in all ASD age groups (Gap and Overlap latency), our results suggest that while speed of processing for basic sensorimotor processing is intact, speed of processing and response preparation for executive control of behavior is abnormal in autism [55]. In contrast to prosaccade tasks that are basic sensory motor tasks, Antisaccade task engages the same sensory motor regions but requires more effort in motor processing. Results in imagery studies suggested that cortical eye movement control regions such as the FEF were recruited to a higher degree for inhibitory processes in the Antisaccade task, relative to pure sensorimotor processes required in prosaccades tasks. Anomalies in FEF and pre-supplementary motor area (SMA) have been found to impair inhibitory control while keeping simple saccadic processes intact [99]. Previous studies have focused attention on the neural correlates of response inhibition in autism and associated abnormalities in the cortical frontal eye field (FEF) and the anterior cingulate cortex [45, 71, 100], the two key brain areas involved in generating and controlling volitional saccades. The FEF is engaged in the preparation of the antisaccade response, whereas the ACC is activated in conflict monitoring, which in the Antisaccade task is represented by an inhibition of the reflexive saccade toward a peripheral target and the execution of a saccade in the opposite direction [101–104]. The left precuneus and left putamen are specifically involved in ASD compares to non ASD peers [49, 56]. Such difficulties with top-down cognitive control have already been implicated in ASD [100]. The activation of these areas was found to be associated with the presence and expression of repetitive behaviors in ASD, suggesting that frontostriatal dysfunction may underlie behavioral rigidity [39].

These findings are also consistent with various neuropsychological studies, comparing executive function and attention-shifting abilities in ASD, which have found no evidence for difficulties in reflexive shifting of attention in autism, but have shown consistent evidence for atypicalities in the higher order voluntary regulation of attentional focus [105].

These atypical responses in ASD could be explained by a more globally impaired cognitive control of behavior, reflecting an impaired capacity of the prefrontal cortex to volitionally suppress context-inappropriate reflexive saccades [24, 52, 55, 56, 71]. If individuals with ASD show parallel improvements in inhibitory control through development, with decreasing errors rates and increasing gain, they do not catch up with typically developing peers.

Consistent with this, clinical executive dysfunctions in inhibition (BRIEF-BRI subscores) were correlated with speed and accuracy measures in this oculomotor task and with erroneous saccades rates in our data.

## Limitations

Despite the novelty of this study in its investigation of separate age groups using the same evaluation protocols over a 2-year follow-up period, several limitations should be noted. First, the sample size of each developmental group was relatively small and in particular the TD groups, with a resultant effect on the significance levels of some data. Therefore, future studies should include larger number of participants in addressing developmental issues. Second, we analyzed TD oculomotor behavior solely at the T0 time point. Future follow-up studies should include TD participant groups at later time points in order to directly compare the maturational dynamics of executive functions in both ASD and TD participants. Third, no ADHD control group was involved in our study. In order to more precisely test the impact of the comorbidity of this condition with ASD on oculomotor measures, the inclusion of an ADHD participant group without ASD symptoms should also be considered. Fourth, an interpretative bias in our results may have arisen from our age grouping, particularly the teenager group which extended from 12 to 18 year old, i.e., effectively from old children to young adults. Moreover, due to their restricted sample population size, the teenager and child groups were not matched for gender. This could be responsible for a bias in group comparison, as gender differences in executive functions and specifically in impulsivity have been already reported in the general population. Various results indicate faster reaction times, higher rates of premature responses and impulsivity, in boys than in girls [for review, see 104], even if differences in ability in executive functions may in fact reflect different strategies.

## Conclusions

In this study, different lines of evidence were found in favor of atypical attention disengagement and attention switching, atypical inhibition and of impulsivity in high functioning ASD participants. Abnormalities in prosaccade tasks were revealed solely by accuracy deficits (gain) and a lack of inhibition with higher erroneous saccade rates. Latencies in prosaccade tasks did not differ in ASD from TD peers except in adolescence stage and appear to follow a similar developmental progression, in contrast to latencies in the Antisaccade task. The results from this task provided evidence for different developmental trajectories between ASD and TD individuals with respect to inhibition skills.

Based on the observation of an age-dependent general improvement in oculomotor skills, which occurs in ASD subjects for accuracy and global functions in prosaccades tasks but not the Antisaccade task, our results support the conclusion that a specific developmental dysfunction occurs in the maturation of inhibition and executive functions in ASD. However, as in TD individuals, attention and inhibition functions in ASD improve during the course of development, and even over the 2-year period of our follow-up study, an improvement in accuracy was observed in children with ASD.

Our data may explain several contradictory findings in the literature. They should also drive future training and therapeutic approaches that target attention processing and inhibitory control in ASD without intellectual disability, by (1), accounting for the age of subjects, (2), separating ADHD comorbidity effects and specific inhibitory atypicalities due to ASD, which seem to improve naturally with age, and (3) helping to prevent misinterpreting the potential effect of training and/or therapeutics as alone being responsible for any significant improvement of attentional and inhibitory processing observed in follow-up monitoring of ASD children. Future research coupling eye tracking, neuroimaging, autistic symptomatology and daily functioning evaluations are now needed, along with the integration of age and ADHD comorbidity biases, to explore the relationships between reflexive and voluntary saccade measures and cortical network dysfunctionality—especially in cerebello-thalamo-cortical pathways—in ASD and to better understand the clinical impact of these disorders.

## Supplementary Information

**Additional file 1**. Oculomotor measures in ASD and TD groups for each 4 task and for each variable at TO. Mean values ± sem.

**Additional file 2**. Difference (delta) in latency values for Gap effect (Step-Gap), Overlap effect (Overlap-Step) and Antisaccade effect (Antisaccade-Gap) at TO. A: Adults; C: Children; T: teenagers; Mean values ± sem.

**Additional file 3**. Difference (delta) in latency values for Gap effect (Step-Gap), Overlap effect (Overlap-Step) and Antisaccade effect (Antisaccade-Gap over the two year follow up. T0: time zero (onset of the study); Y1: year 1; Y2: year 2; Mean values ± sem.

## Data Availability

The data sets used and/or analyzed during the study are available on request from the corresponding author.

## Abbreviations

ACC: Anterior cingulate cortex
ADHD: Attention-deficit/hyperactivity disorder
ADHD-RS IV: Attention-Deficit/Hyperactivity Disorder Rating Scale
ADI-R: Autism Diagnostic Interview-Revised
ADOS: Autism Diagnostic Observation Schedule
All ASD: Children, teenagers and adults with ASD
All Adults: ASD and TD adult participants
All Children: ASD and TD children participants
All TD: TD children, teenagers and adults
All Teenagers: ASD and TD teenager participants
ANOVA: Analysis of variance
AOIs: Areas of interests
ASD: Autism spectrum disorder
BRIEF: Behavior rating inventory of executive function
DIGS: Diagnostic interview for genetics studies
DSM-5: Diagnostic and statistical manual of mental disorders
F: Female
FEF: Frontal eye field
IQ: Intelligence quotient
K-SADS: Kiddie schedule for affective disorders and schizophrenia
M: Male
N: Number
RT: Reaction time
SD: Standard deviation
SEM: Standard error of the mean
T0: Time point of first assessment
TD: Typically developing
VGS: Visually guided saccades
WAIS-III or -IV: Wechsler Adult Intelligence Scale
WISC-IV: Wechsler Child Intelligence Scale
Y1: 12 Months after T0
Y2: 24 Months after T0
