# Correction to: Visual attention and inhibitory control in children, teenagers and adults with autism without intellectual disability: results of oculomotor tasks from a 2-year longitudinal follow-up study (InFoR)

**DOI:** 10.1186/s13229-021-00479-x

**Published:** 2022-01-05

**Authors:** Anouck Amestoy, Etienne Guillaud, Giulia Bucchioni, Tiziana Zalla, Daniel Umbricht, Christopher Chatham, Lorraine Murtagh, Josselin Houenou, Richard Delorme, Myriam Ly-Le Moal, Marion Leboyer, Manuel Bouvard, Jean-René Cazalets

**Affiliations:** 1 CNRS, Aquitaine Institute for Cognitive and Integrative Neuroscience, INCIA, UMR 5287, Université de Bordeaux, 33000 Bordeaux, France; 2 iBrain, UMR 1253 Inserm, Université de Tours, 2 Boulevard Tonnellé, 37044 Tours Cedex, France; 3 Roche Pharma Research and Early Development, Roche Innovation Center Basel, F. Hofmann-La Roche Ltd., Grenzacherstrasse 124, 4070 Basel, Switzerland; 4 Laboratoire de NeuroPsychiatrie Translationnelle, INSERM, U955, IMRB, Créteil, France; 5 Fondation FondaMental, Créteil, France; 6 NeuroSpin, UNIACT Lab, Equipe de Psychiatrie, Commissariat À L’énergie Atomique, Saclay, Gif-sur-Yvette, France; 7 Institut Pasteur, Paris, France; 8 AP-HP, DMU IMPACT, Psychiatry and Addictology Department, Mondor University Hospital, Université Paris Est Créteil, Créteil, France; 9 Institut Roche, Tour Horizons-Bureau 18M3, Roche S.A.S., 30, cours de l’île Seguin, 92650 Boulogne-Billancourt, France; 10 Centre Hospitalier Charles-Perrens, Pôle Universitaire de Psychiatrie de L’enfant Et de L’adolescent, 121, rue de la Béchade, CS 81285, 33076 Bordeaux Cedex, France; 11 Institut Jean Nicod, ENS, Paris, France

## Correction to: Molecular Autism (2021) 12:71 10.1186/s13229-021-00474-2

Following publication of the original article [1], the authors identified 2 errors:

1. An incorrect author name
  - **Incorrect name**: Miriam Ly-Le Moal
  - **Correct name**: Myriam Ly‐Le Moal
2. The wrong affiliation was listed for Tiziana Zalla
  - **Incorrect affiliation**: Roche Pharma Research and Early Development, Roche Innovation Center Basel, F. Hofmann-La Roche Ltd., Grenzacherstrasse 124, 4070 Basel, Switzerland.
  - **Correct affiliation**: Institut Jean Nicod, ENS, Paris

The author group has been updated above, and the original article [1] has been corrected.
