# Supplementary material for: Visual attention and inhibitory control in children, teenagers and adults with autism without intellectual disability: results of oculomotor tasks from a 2-year longitudinal follow-up study (InFoR)
Source: Mol Autism. 2021 Nov 13;12:71. doi: 10.1186/s13229-021-00474-2 (PMC8590241; doi:10.1186/s13229-021-00474-2)
Supplement: Supplementary file 1 — Additional file 1. Oculomotor measures in ASD and TD groups for each 4 task and for each variable at TO. Mean values ± sem. [file 13229_2021_474_MOESM1_ESM.docx]

**Additional file 1** **:** Oculomotor measures in ASD and TD groups for each 4 task and for each variable at TO. Mean values ± sem.

| ***Variables*** | **TASKS** | | | | |
| --- | --- | --- | --- | --- | --- |
| ***Latency (ms)*** |  | **GAP** | **STEP** | **OVERLAP** | **ANTISACCADE** |
| **Children** | **TD (9)** | 327.79 ± 20.17 | 398.65 ± 9.16 | 449.71 ± 30.76 | 551.57 ± 34.04 |
|  | **ASD(33)** | 316.07 ± 8.98 | 384.59 ± 7.56 | 452.55 ± 19.21 | 405.18 ± 15.59 |
|  | **All (42)** | 318.82 ± 8.19 | 387.67 ± 6.25 | 451.82 ± 16.16 | 445.10 ± 17.37 |
| **Teenagers** | **TD (11)** | 329.13 ± 14.1 | 387.08 ± 10.98 | 475.53 ± 22.32 | 553.49 ± 22.92 |
|  | **ASD (29)** | 340.19 ± 12.49 | 363.58 ± 7.76 | 424.24 ± 16.58 | 414.30 ± 7.4 |
|  | **All (40)** | 337.03 ± 9.72 | 370.87 ± 6.49 | 439.13 ± 13.79 | 452.26 ± 12.78 |
| **Adults** | **TD (29)** | 371.21 ± 9.1 | 401.42 ± 8.19 | 460.99 ± 15.34 | 477.34 ± 11.95 |
|  | **ASD (35)** | 372.60 ± 10.02 | 399.63 ± 9.54 | 464.61 ± 16.34 | 474.86 ± 17.95 |
|  | **All (64)** | 371.97 ± 6.8 | 400.44 ± 6.34 | 462.90 ± 11.19 | 476.10 ± 10.69 |
| ***Erroneous saccade percent (%)*** |  | **GAP**  (MEAN± SEM) | **STEP**  (MEAN± SEM) | **OVERLAP**  (MEAN± SEM) | **ANTISACCADE**  (MEAN± SEM) |
| **Children** | **TD** | 16.13 ± 3.44 | 8.25 ± 3.05 | 7.04 ± 3.7 | 25.27 ± 7.46 |
|  | **ASD** | 17.42 ± 2.09 | 3.99 ± 1.15 | 5.71 ± 1.33 | 33.28 ± 3.65 |
|  | **All** | 17.10 ± 1.77 | 5.03 ± 1.14 | 6.05 ± 1.31 | 31.34 ± 3.28 |
| **Teenagers** | **TD** | 12.22 ± 3.77 | 0.00 ± 00 | 0.91 ± 0.87 | 8.37 ± 4.31 |
|  | **ASD** | 18.22 ± 3.36 | 6.51 ± 1.62 | 9.47 ± 1.81 | 27.27 ± 4.44 |
|  | **All** | 16.34 ± 2.62 | 4.68 ± 1.25 | 6.88 ± 1.45 | 22.37 ± 3.66 |
| **Adults** | **TD** | 9.88 ± 1.52 | 3.72 ± 1.29 | 4.12 ± 1.37 | 10.47 ± 2.34 |
|  | **ASD** | 11.97 ± 2.2 | 6.02 ± 1.76 | 4.07 ± 1.7 | 17.65 ± 3.25 |
|  | **All** | 10.98 ± 1.37 | 4.98 ± 1.13 | 4.09 ± 1.11 | 14.36 ± 2.1 |
| ***Anticipatory saccade percent (%)*** |  | **GAP**  (MEAN± SEM) | **STEP**  (MEAN± SEM) | **OVERLAP**  (MEAN± SEM) | **ANTISACCADE**  (MEAN± SEM) |
| **Children** | **TD** | 5.01 ± 1.75 | 4.36 ± 1.87 | 8.01 ± 3.14 | 11.87 ± 4.68 |
|  | **ASD** | 11.00 ± 2.5 | 9.76 ± 2.14 | 6.63 ± 1.51 | 9.38 ± 2.13 |
|  | **All** | 9.54 ± 2 | 8.44 ± 1.73 | 6.98 ± 1.35 | 9.98 ± 1.93 |
| **Teenagers** | **TD** | 5.25 ± 2.58 | 1.48 ± 1.34 | 0.91 ± 0.87 | 2.04 ± 1.63 |
|  | **ASD** | 13.73 ± 2.6 | 5.97 ± 1.48 | 9.38 ± 2.45 | 6.57 ± 1.71 |
|  | **All** | 11.08 ± 2.6 | 4.70 ± 1.17 | 6.81 ± 1.86 | 5.40 ± 1.35 |
| **Adults** | **TD** | 8.76 ± 3.01 | 5.75 ± 2.88 | 4.57 ± 2.28 | 4.14 ± 2.2 |
|  | **ASD** | 7.78 ± 1.51 | 12.94 ± 3.01 | 4.99 ± 1.63 | 5.81 ± 1.79 |
|  | **All** | 8.25 ± 1.6 | 9.69 ± 2.13 | 4.80 ± 1.36 | 5.04 ± 1.39 |
| ***Gain first fixation*** |  | **GAP**  (MEAN± SEM) | **STEP**  (MEAN± SEM) | **OVERLAP**  (MEAN± SEM) | **ANTISACCADE**  (MEAN± SEM) |
| **Children** | **TD** | 0.76 ± 0.04 | 0.81 ± 0.03 | 0.84 ± 0.02 | 0.59 ± 0.06 |
|  | **ASD** | 0.73 ± 0.02 | 0.80 ± 0.02 | 0.80 ± 0.02 | 0.65 ± 0.02 |
|  | **All** | 0.73 ± 0.02 | 0.80 ± 0.01 | 0.81 ± 0.02 | 0.63 ± 0.02 |
| **Teenagers** | **TD** | 0.83 ± 0.04 | 0.89 ± 0.02 | 0.89 ± 0.03 | 0.81 ± 0.05 |
|  | **ASD** | 0.82 ± 0.03 | 0.83 ± 0.02 | 0.88 ± 0.02 | 0.68 ± 0.03 |
|  | **All** | 0.82 ± 0.02 | 0.84 ± 0.02 | 0.88 ± 0.02 | 0.72 ± 0.02 |
| **Adults** | **TD** | 0.93 ± 0.01 | 0.94 ± 0.01 | 0.95 ± 0.01 | 0.83 ± 0.03 |
|  | **ASD** | 0.89 ± 0.02 | 0.91 ± 0.02 | 0.89 ± 0.02 | 0.76 ± 0.02 |
|  | **All** | 0.91 ± 0.01 | 0.92 ± 0.01 | 0.92 ± 0.01 | 0.79 ± 0.02 |
