# Supplementary material for: Visual attention and inhibitory control in children, teenagers and adults with autism without intellectual disability: results of oculomotor tasks from a 2-year longitudinal follow-up study (InFoR)
Source: Mol Autism. 2021 Nov 13;12:71. doi: 10.1186/s13229-021-00474-2 (PMC8590241; doi:10.1186/s13229-021-00474-2)
Supplement: Supplementary file 2 — Additional file 2. Difference (delta) in latency values for Gap effect (Step-Gap), Overlap effect (Overlap-Step) and Antisaccade effect (Antisaccade-Gap) at TO. A: Adults; C: Children; T: teenagers; Mean values ± sem. [file 13229_2021_474_MOESM2_ESM.docx]

**Additional file 2** : Difference (delta) in latency values for Gap effect (Step-Gap), Overlap effect (Overlap-Step) and Antisaccade effect (Antisaccade-Gap) at TO. A : Adults ; C, Children ; T ; teenagers ; Mean values ± sem.

|  |  | **Delta STEP-GAP**  *(GAP Effect)* | **Delta OVERLAP-STEP**  *(OVERLAP Effect)* | **Delta GAP-OVERLAP**  *(GAP-OVERLAP Effect)* | **ANTISACCADE Effect** |
| --- | --- | --- | --- | --- | --- |
| **C** | TD | 75.6 ± 14.31 | 48.53 ± 32.68 | 97.45 ± 42.11 | 175.08 ± 27.1 |
|  | ASD | 68.04 ± 8.84 | 50.26 ± 11.56 | 148.76 ± 20.03 | 90.57 ± 17.15 |
|  | ALL | 44.7644 ± 9.9 | 49.81 ± 11.38 | 134.77 ± 18.29 | 112.81 ± 15.39 |
| **T** | TD | 64.75 ± 13.31 | 98.94 ± 18.32 | 172.5 ± 25.4 | 201.35 ± 24.35 |
|  | ASD | 36.35 ± 12.62 | 47.45 ± 10.63 | 60.4 ± 18.33 | 71.76 ± 9.9 |
|  | ALL | 44.76 ± 9.9 | 62.70 ± 9.79 | 89.45 ± 16.78 | 97.68 ± 12.35 |
| **A** | TD | 23.09 ± 9.8 | 51.91 ± 12.42 | 94.132 ± 13.34 | 92.59 ± 13.08 |
|  | ASD | 30.87 ± 8.59 | 51.49 ± 11.87 | 85.77 ± 14.85 | 117.66 ± 18.24 |
|  | ALL | 27.20 ± 6.42 | 51.68 ± 8.5 | 89.8 ± 11.9 | 105.84 ± 11.52 |
