# Supplementary material for: Visual attention and inhibitory control in children, teenagers and adults with autism without intellectual disability: results of oculomotor tasks from a 2-year longitudinal follow-up study (InFoR)
Source: Mol Autism. 2021 Nov 13;12:71. doi: 10.1186/s13229-021-00474-2 (PMC8590241; doi:10.1186/s13229-021-00474-2)
Supplement: Supplementary file 3 — Additional file 3. Difference (delta) in latency values for Gap effect (Step-Gap), Overlap effect (Overlap-Step) and Antisaccade effect (Antisaccade-Gap over the two year follow up. T0: time zero (onset of the study); Y1: year 1; Y2: year 2; Mean values ± sem. [file 13229_2021_474_MOESM3_ESM.docx]

**Additional file 3** : Difference (delta) in latency values for Gap effect (Step-Gap), Overlap effect (Overlap-Step) and Antisaccade effect (Antisaccade-Gap over the two year follow up. T0 : time zero (onset of the study) ; Y1 : year1 ; Y2 : year 2 ; Mean values ± sem.

|  | GAP Effect | | | OVERLAP Effect | | | ANTISACCADE Effect | | |
| --- | --- | --- | --- | --- | --- | --- | --- | --- | --- |
|  | T0 | Y1 | Y2 | T0 | Y1 | Y2 | T0 | Y1 | Y2 |
| C | 76.28 ± 10.94 | 56.53 ± 15.83 | 36.9 ± 10.88 | 50.26 ± 11.56 | 38.41 ± 6.6 | 67.45 ± 12.8 | 90.57 ± 17.15 | 121.76 ± 25.27 | 124.7 ± 25.7 |
| T | 36.35 ± 12.62 | 50.39 ± 9.87 | 72.12 ± 11.55 | 47.45 ± 10.63 | 32.09 ± 7.59 | 10.72 ± 11.74 | 71.76 ± 9.9 | 79.16 ± 16.37 | 112.07 ± 15.18 |
| A | 30.87 ± 8.59 | 18.77 ± 10.39 | 26.49 ± 7.15 | 51.49 ± 11.87 | 48.56 ± 8.24 | 35.38 ± 7.95 | 109.29 ± 16.95 | 46.83 ± 13.96 | 107.6 ± 14.9 |
